# Plastics in Total Knee Replacement: Processing to Performance

**DOI:** 10.7759/cureus.12969

**Published:** 2021-01-28

**Authors:** Naga Cheppalli, Sreenivasulu Metikala, Benjamin S Albertson, Kenneth Yaw

**Affiliations:** 1 Orthopaedics, Veteran Affairs (VA) Hospital/University of New Mexico Hospital, Albuquerque, USA; 2 Orthopaedics, Virginia Commonwealth University Health System, Richmond, USA; 3 Orthopaedics & Rehabilitation, University of New Mexico Health Sciences Center, Albuquerque, USA; 4 Orthopaedics, New Mexico Veteran Affairs (VA) Health Care System, Albuquerque, USA

**Keywords:** polyethylene, crosslinked, knee arthroplasty, wear, sterilization, packaging, oxidation, fatigue, antioxidant, vitamin e

## Abstract

Polyethylene (PE) is the key component of total knee replacement (TKR). The wear of polyethylene, a common cause of revision surgeries, depends on multiple factors. The mechanical properties, wear characteristics, and oxidative resistance of PE can be manipulated by the techniques of processing, sterilization, and packaging methods. This article describes the making of conventional and cross-linked poly, packaging, sterilization, processing techniques, and a summary of commercially available plastics and their rationale in TKR including the latest advances.

## Introduction and background

Polyethylene (PE) is an integral part of total knee replacement (TKR) and has undergone significant changes in the last three decades. PE wear is one of the common causes of revision knee replacement [1-3]. Multiple factors predispose PE to wear. Some of them are methods of PE processing (packaging, sterilization), type of implants, the thickness of the PE, locking mechanisms, surface roughness of the metallic tibial tray, surface contour of PE and area of articulation with the femoral component, types of movements between the bearing surfaces (rolling, sliding, and rotational motion), presence of third body wear, alignment inaccuracies and patient-related factors like body mass index (BMI), and patient activity level [4,5]. Further, various PE designs such as cruciate-retaining, posterior-stabilized, medial congruent, medial pivot, anterior stabilized, constrained poly with a longer and wider post, bicruciate stabilized components can influence the mechanical forces transmitted at the area of contact and contribute to wear [6,7].

This article focuses on 1) processing of PE, 2) packaging and sterilization techniques, 3) mechanical and wear properties of the final product, and 4) product options available from different implant manufacturers.

## Review

Making of conventional polyethylene (CPE)

Raw Material

PE is a polymer of ethylene consisting of as many as 200,000 ethylene repeat units. Ethylene is polymerized in the presence of catalysts to make ultra-high molecular weight polyethylene (UHMWPE), which is commercially produced as resin powder. There are three types of resins labeled as GUR 1020 (Type 1), GUR 1050 (Type 2), and 1900 H (Type 3). This classification is based on the presence of impurities such as titanium, aluminum, chlorine, calcium, as well as storage and handling properties. The acronym GUR stands for Granular-UHMWPE-Ruhrchemie, which came from Celanese, the first company to manufacture UHMWPE for Orthopedics. Currently, GUR 1020 and GUR 1050 powders are routinely used, although most manufacturers prefer the former type because of its higher ductility and impact strength. Since 2002, the resin 1900 H (type 3) has been withdrawn from the market.

Processing

**Figure 1:**
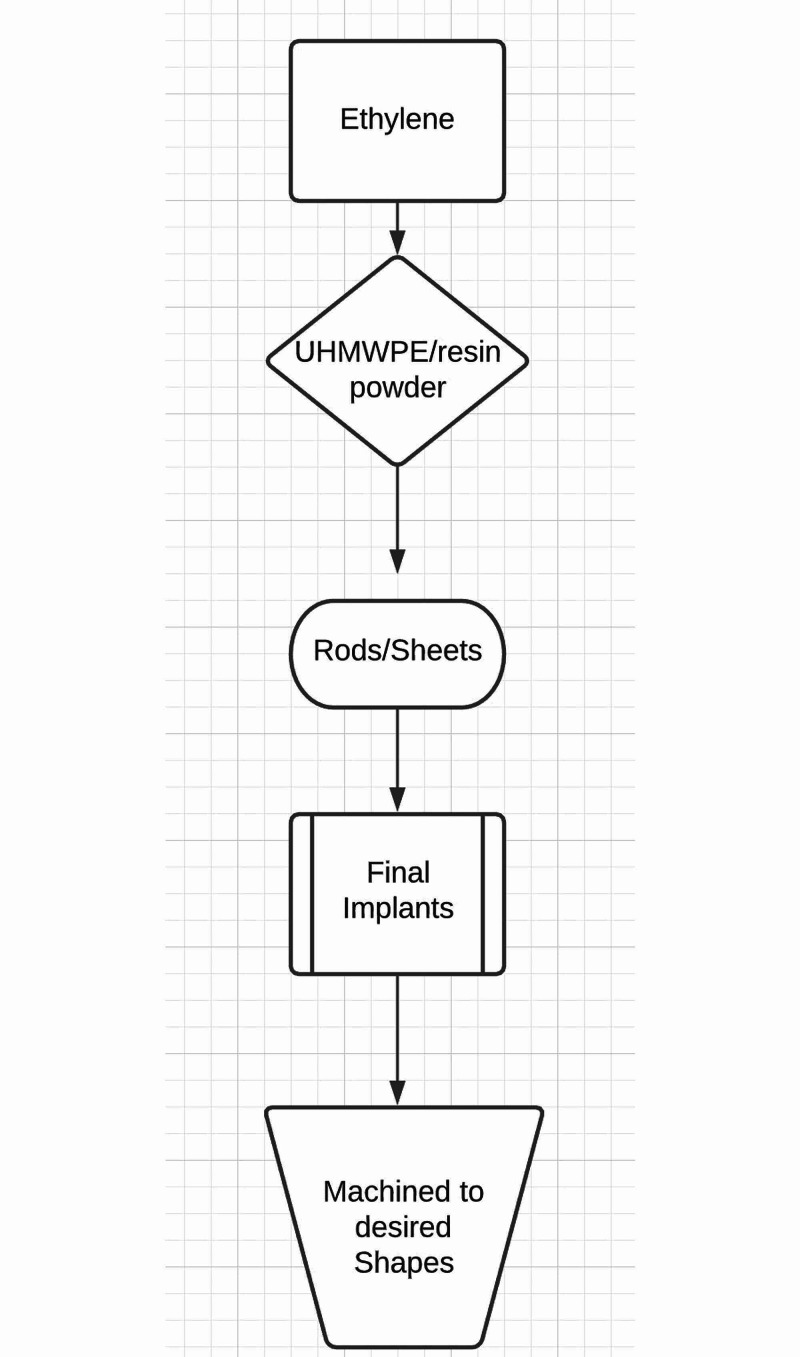
Flow diagram of the making of conventional polyethylene (CPE)

The chosen resin powder is consolidated into rods/sheets from which final implants are made. Historically, this conversion is done by compression molding (popular in Europe), ram extrusion (popular in the United States), hot isostatic pressing (used by Biomet, Inc, Warsaw, IN), or direct compression molding (DCM), which is also known as net shape compression molding where the resin powder is directly converted into the finished or semi-finished product [8]. Unfinished products are machined into the desired shapes (Figure 1).

Non-articulating surfaces of the components are machined to accommodate the locking mechanism. Machining, however, may cause microscopic surface irregularity, which can contribute to future wear. Therefore, the speed of milling devices and the heat produced during the machining process are highly regulated to minimize deteriorating effects on PE. Thus, the resin type, conversion method, and machining to the final product have individual effects on the physical properties and wear characteristics of the final plastic product. Since there has been no consensus to determine the best plastic production process in the orthopedic industry, the implant companies have sought their own strategies for processing UHMWPE.

Packaging and Sterilization

Once final implants are made, packaging and sterilization of PE are the crucial steps that can significantly affect PE's wear properties. Basically, there are five main methods of sterilization (Table 1).

**Table 1:** Summary of various sterilization processes Mrd: megarads

Sterilization Process	Packaging Type	Radiation Dose (Mrd)
Gamma air	Gas permeable	2.5-4.0
Gamma inert	Oxygen barrier packaging/ reduced oxygen atmosphere	2.5-4.0
Electron beam (E-beam)	Reduced oxygen atmosphere	2.5-4.0
Ethylene Oxide	Gas permeable	None
Gas plasma	Gas permeable	None

Radiation techniques include gamma irradiation and electron beam (E-beam). Ethylene Oxide (EtO) and gas plasma (GP) are the non-radiation sterilization methods. In the past, sterilization was done by gamma irradiation in the presence of air. This method, however, had resulted in the production of innumerable free radicals that typically react with oxygen causing PE chain scission and significant oxidative wear. In an attempt to minimize such free radical production, sterilization has now been performed in the absence of air or near-vacuum (Depuy Orthopedics, Warsaw, IN) or in the presence of inert gases like Nitrogen (Stryker Orthopedics, Mahwah, NJ and Zimmer, Inc, Warsaw, IN) or Argon (Biomet). Also, the final plastic is wrapped using barrier packaging materials which in turn helps to prevent oxidation. However, PE chain breakage can still happen in the long-term because of the production of residual free radicals which are responsible for ongoing oxidative damage. The generation of such a small quantity of free radicals is inevitable despite meticulous processing techniques, which is considered as an acceptable minimum level. EtO is an alternative sterilization method to radiation, which totally eliminates oxidative damage. While this method can better preserve all the properties of PE, handling EtO is expensive and time-consuming. Recently, GP sterilization is another alternative that relies on ionized gas. It has been showing promising results without leaving any toxic residues. Surgeons should be aware that the shelf life of PE depends on the methods of packaging and sterilization. At this time, there is no consensus in the United States regarding the acceptable shelf life. Whereas in Europe, a standard practice of five years of shelf life has been adopted [9]. Given that sterilization by EtO or GP does not produce any free radicals, the longer shelf life of more than five years may be justifiable. An irradiated plastic either by gamma or E-beam necessitates gas barrier packaging methods to prevent oxidation, while gas permeable packaging is acceptable for EtO and GP sterilization techniques [10].

Cross-linked polyethylene (XLPE)

**Figure 2:**
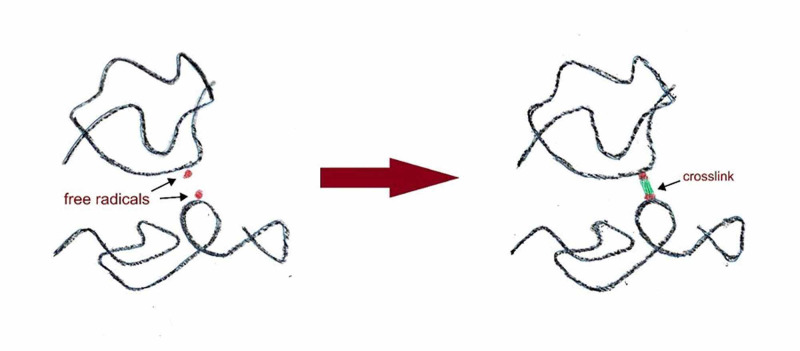
The process of cross-linking of adjacent chains

The next successful attempt to improve the mechanical properties of PE, particularly the wear resistance, is achieved by cross-linking adjacent chains through higher doses of irradiation, producing XLPE (Figure 2).

Initially, cross-linking was induced by a single sterilization dose (2.5 to 4.0 Mrd), which substantially enhanced wear performance. However, it led to a reduction in fatigue crack propagation (FCP) resistance, ultimate tensile strength, elongation at break values, and impact toughness [10]. The XLPE plastics have been in widespread use since the late 1990s [11]. Further increasing the radiation dose (5 to 10 Mrd), results in radical species that react with chain imperfections leading to polymer chain cross-linking with stable C-C chemical bonds. It theoretically increases the molecular mass to infinity, thus producing highly cross-linked polyethylene (HXLPE), which came to the market in 1998 [8].

Both gamma and E-beam radiation are used to cross-link PE. Gamma radiation generally uses Cobalt-60 as the source of radiation. While gamma radiation can penetrate through resin powder, the penetration of E-beam radiation is limited by its kinetic energy [9]. Nonetheless, there is no significant difference in the formation of macro radicals between these two methods. Although higher radiation doses can significantly improve wear resistance, it results in brittleness of plastic and poor mechanical properties. Also, the wear particles generated from HXLPE can be smaller and biologically more active than non-irradiated ones [12,13]. Thermal treatment, either by remelting or annealing, can improve some of these characteristics, as described below.

Thermal Treatment - Remelting

In an effort to absorb residual free radicals and improve oxidation properties, the irradiated HXLPE is remelted above its melting temperature (greater than 150 degrees Celsius), which is typically done before the plastic is machined into the final product. Although such post-irradiation remelting likely increases the long-term oxidation resistance, it further compromises the mechanical properties due to the microstructure's alteration, decreased crystallinity, and low FCP resistance [14]. Few cases of tibial post fractures have been reported with remelted PE [15].

Thermal Treatment - Annealing

Annealing has been introduced to impart higher mechanical properties, where reheating of HXLPE is done close to but below its melting temperature. This process retains the crystallinity of plastic and improves fatigue performance while preserving the wear characteristics [5]. However, the annealed plastic has not proven to be as effective as the remelted poly in terms of reducing the quantity of residual free radicals. Consequently, this led to increased oxidative wear and overall deterioration of mechanical properties when subjected to the accelerated aging test. For these reasons, the annealed PE never took off commercially.

Sequentially Irradiated and Annealed Polyethylene

It is a unique process where compression-molded GUR 1020 resin undergoes sequential irradiation and annealing to produce a particular type of HXLPE, the X3 (Stryker Orthopaedics, Mahwah, NJ). The process of sequential irradiation happens in three different cycles, with each cycle set at a radiation dose of 3 Mrd followed by annealing for 8 hours at 130 degrees C. Following this process, the material's outer 1-3 mm is removed, the component is then machined and packaged for GP sterilization. The cumulative radiation dose thus becomes 9 Mrd. The X3 is designed to retain mechanical properties while reducing the proportion of free radicals. X3 poly was initially introduced for hips in 2005, followed by knees in 2008. A knee simulator study comparing six poly designs demonstrated the lowest wear rates with X3 poly [16]. However, early failures with fractures and oxidation damage have been reported with this design affecting mainly the posteromedial and lateral borders [17]. Although there are no long term data using X3 in TKA, the short term studies have demonstrated superior wear characteristics [18,19].

HXLPE can be classified into first and second generation based on processing technique. When subjected to post-irradiation thermal treatment, it is considered the first generation. In comparison, sequential irradiation-annealing or addition of antioxidants is considered to be the second-generation category.

Antioxidants

To overcome long-term oxidation while preserving the mechanical properties of HXLPE, the manufacturers have begun the addition of antioxidants, specifically Vitamin E (VitE), a natural antioxidant that has been used in food packaging since 1980 [8]. There are two methods of incorporating VitE. The first method includes blending VitE into resin powder (in concentrations ranging from 1000-8000 ppm) even before consolidation and irradiation. Studies demonstrated that this method prevents in-vivo oxidation, accelerated aging, and improves wear characteristics but at the expense of decreased cross-linking efficiency [20]. For that reason, an alternative method of VitE diffusion (soaking or infusion) into poly following radiation has been introduced.

VitE-coated blocks are placed into an inert oven and homogenized (baked) at 120°C until the vitamin diffuses through the block's thickness. This technique resulted in higher oxidation resistance in vitro, along with ultra-low wear, and retained mechanical strength. There is a theoretical concern that with progressive wear, VitE may elute into the joint and get absorbed systemically. To prevent such elution, VitE is bonded to PE by covalent bonds (grafting). Even in this hypothetical situation, the amount of VitE that diffuses into the system is thought to be too low to cause any cellular cytotoxic effects. In an in vitro study, VitE poly demonstrated decreased bacterial adherence (Staphylococcus epidermis and S. aureus) [21,22]. Overall, preclinical studies have shown promising improvements in fatigue strength when doped with VitE while maintaining the wear properties and oxidation resistance comparable to irradiated and remelted HXLPE [23]. In vitro studies showed that VitE poly has five times greater ultimate strength after four weeks of accelerated aging test and up to an 86% lower wear rate than CPE [24]. Further, lower rates of bone resorption and inflammatory fibrous tissue were noted with VitE poly than HXLPE [25,26]. In literature, fracture of VitE poly has been reported in THR but not in TKR [27]. However, there are no midterm or long-term studies available on the use of VitE poly in TKR.

Table 2 illustrates the evolution of plastics and the differences in their characteristics. 

**Table 2:** Evolution of plastics and their characteristics ^a ^cannot prevent in-vivo oxidation ^b^ can prevent long term in-vivo oxidation CPE: conventional polyethylene; XLPE: cross-linked polyethylene; HXLPE: highly cross-linked polyethylene; Mrd: megarads.

	Wear resistance	Mechanical properties	Oxidation resistance	Mechanical properties after aging	Osteolysis potential from a wear particle	X-linking dose	Manufacturers
CPE	+	+++	++	++	+	None	Medacta; Microport; Exachtech; Smith & Nephew (only for constrained poly)
Moderate XLPE	++	++	+	+	++	< 5 Mrd	N2 VAC (Stryker)
Remelted HXLPE (1^st^ generation)	+++	+	++^a^	+	++	5-7.5 Mrd	Durasul (Zimmer); Prolong (Zimmer); XLPE (Smith &Nephew)
Annealed HXLPE (1^st^ generation)	+++	++	+	+	++	5-7.5 Mrd	Not available
Sequentially irradiated and annealed HXLPE (2^nd^ generation)	+++	+++	+++^ a^	++	+	9 Mrd	X3 (Stryker)
Addition of antioxidants	+++	+++	+++^b^	+++	+	Up to 10 Mrd	E1 poly (Biomet); Vivacet (Zimmer); AOX (Depuy)

Using CPE for TKA

While most companies have adopted newer technology, some still market CPE (non-cross linked) due to the concerns that cross-linking leads to inferior mechanical properties. Thermal treatment of irradiated HXLPE further compromises the mechanical properties. As discussed earlier, remelted HXLPE has good oxidation and wear performances but possesses reduced crystallinity and lower fatigue strength. On the other hand, annealed HXLPE has fair wear and fatigue performances but poor oxidation resistance [28,29]. Further, it has been demonstrated that wear particles induced by HXLPE and CPE have different biological host responses [30-32]. In an animal model, a greater risk of particle-induced osteolysis and heightened inflammatory response was observed in the HXLPE group compared to CPE and VitE poly [12]. Finally, the proponents of CPE argue that the mode of poly failure is different in THA compared to TKA. The most common cause of PE failure in TKA is delamination [33] while adhesive or abrasive wear is the common cause in THA due to better conformity and even stress distribution [16,33]. For these reasons, some manufacturers prefer the preservation of mechanical properties to wear properties in an attempt to avoid such delamination.

Table 3 depicts a summary of various commercially available plastics & their processing methods for TKR.

**Table 3:** Summary of commercially available plastics for total knee replacement VitE: vitamin E; Mrd: megarad; EtO: ethylene oxide; XLPE: cross-linked polyethylene; N/A: not available.

Manufacturer	Plastic name	Resin	Processing	Sterilization	Packaging
Zimmer (Persona)	Vivacet-E	GUR 1020	10 Mrd; E-beam at elevated temperature; No further heat treatment; Vit E is pre-blended with resin	EtO	Air
Zimmer (Nexgen)	Prolong	GUR 1020 GUR 1050	6.5 Mrd; E-beam at elevated temperature; Remelted	Gas plasma/ EtO	Nitrogen/ High oxygen barrier
Zimmer (Natural Knee 2)	Durasul	GUR 1050	9.5 Mrd; E-beam; Remelted	EtO	Nitrogen
Biomet (Vanguard)	E1 Poly	GUR 1020	10 Mrd; Gamma radiation; No heat treatment; VitE infused after cross-linking	3 Mrd; Gamma radiation in Argon	Argon flushed/ Near-vacuum sealed
Biomet	Arcom XL	GUR 1050	5Mrd; Gamma radiation	Gamma radiation in Argon	Inert environment
Biomet	Arcom (R)	GUR 1050	No X-linking; No thermal treatment	Gamma radiation	Inert environment
Stryker (Triathlon)	X3	GUR 1020	Sequential Gamma irradiation at room temp; 3 Mrd x 3 times (total dose: 9 Mrd); Annealed at 30°C after each cycle	Gas plasma	Nitrogen/ Vacuum sealed
Stryker (Scorpio/NRG)	N2VAC	GUR 1020	Conventional; No radiation	3 Mrd; Gamma radiation in N2	Barrier
Depuy (Attune)	AOX	GUR 1020	8.5 Mrd; Gamma radiation at room temperature; No heat treatment; Covernox (Hindered Phenol.075% and few more antioxidants) in resin	3Mrd; Gamma radiation	Vacuum foil
DePuy Sigma	XLK	GUR 1020	5 Mrd: Gamma radiation at room temperature; Remelted at 155°C for 24 hours and then annealed at 120°C for 24 hours	Gamma radiation/ Gas plasma	Vacuum foil
Depuy LCS	XLK	GUR 1020	5 Mrd; Gamma radiation; Remelted	Gas plasma	Vacuum foil
Depuy	GVF	GUR 1020	No X-linking; No thermal treatment	Gamma radiation	Vacuum foil
Smith & Nephew (Journey 1 and 2) (Legion)	XLPE	GUR 1020	7.5 Mrd; Gamma radiation at room temperature; Remelted at 147°C for at least 5 hours	EtO	Barrier
Smith & Nephew (Genesis II)	XLPE	GUR 1050	No radiation; No thermal treatment	Gas plasma	Barrier
Advance medicals Microport	Duramer -1	GUR 1020	No radiation; No thermal treatment	EtO	N/A
Medacta	GMK	GUR 1020	No radiation; No thermal treatment	EtO	Gas permeable
Aesculap	Beta - PE	GUR 1020	X-linking with beta radiation; No heat treatment	N/A	Inert
Conformis	iPoly XE	GUR 1020	10 Mrd; E-beam at elevated temperature; No heat treatment; Mechanically annealed; VitE blended	Gas plasma	N/A
Exachtech	Logic	GUR 1020	6.5 Mrd; E-beam at 125^0^C; Remelted	Gamma radiation	N/A
Arthrex	E-CIMA	GUR 1020	9.5 Mrd; E-beam; VitE blended at raw material state	Gamma radiation	Vacuum foil

Clinical studies

Existing short and midterm studies demonstrate no differences in revision rates between XLPE and CPE in TKA [32,34]. However, one long-term study (Australian joint registry) showed significantly lower revision rates at a 10-year follow-up with XLPE than CPE (3.5% versus 5.8%) [35].

PE particulate studies

Osteolysis depends on the host response to the amount, size, shape, and quality of PE particles generated from wear. More elongated particles in large quantities tend to produce a greater inflammatory response [36,37]. The particle size less than 0.05 µm or longer than 10 µm fails to elicit an inflammatory response [12,38]. The typical size of wear particles in TKA is approximately 1 µm. Notably, the wear particles from irradiated HXLPE are smaller and biologically more active than from non-irradiated ones. However, periprosthetic osteolysis is not commonly observed because of an overall reduction in the quantity of wear production in HXPLE compared to CPE [34,39].

Registry studies

Table 4 summarizes joint replacement registry studies and the revision rates with CPE and HXLPE [35,40-42].

**Table 4:** Registries data and revision rates CPE: conventional polyethylene; HXLPE: highly cross-linked polyethylene; N/A: not available.

Study	Procedures	Revision rate with CPE	Revision rate with HXLPE	Antioxidants infused	Registry
Inacio	62,177	2.2% at 3 years	2.1% at 1.8 years	N/A	Not listed
Paxton	77,084	2.7% at 5 years	3.1% at 5 years	N/A	Kaiser Permanante Total Joint Replacement Registry
De Steiger	386,104	5.8% at 10 years	3.5% at 10 yr	N/A	Australian Orthopedic Association National Joint Replacement Registry
Partridge	550,658	0.29 aseptic revisions per 100 component years	0.38 aseptic revisions per 100 component years	N/A	National Joint Registry (NJR) for England, Wales, and Northern Ireland 2003-14

Cost

The implant's cost is not standardized and therefore varies significantly among different hospitals, geographic regions, and manufacturers. Given these constraints, it is not easy to pinpoint the exact cost difference between HXLPE and CPE. Data from one hospital suggests that HXLPE inserts cost approximately 150 USD more than CPE, while others estimate this difference to be even higher [43]. With the advent of the bundled payment system in joint replacements, an emphasis has been placed on providing the highest quality care, with quality measured as value/cost. It remains unknown if HXLPE increases the value to TKA sufficient enough to warrant the increased cost compared to CPE. It is still to be determined if it is cost-effective to use HXLPE in only younger patients to avoid future revisions [44].

What's new

A wide range of fillers have been used to enhance PE’s mechanical and lubrication properties. Some of them are carbon nanofibers (CNFs), carbon nanotubes (CNTs), graphene, and hard particles like titanium, zirconium, quartz, natural coral, platinum-zirconium quasicrystal, etc. [45-48]. Surface modifications of PE can also be performed using zirconium carbon nitride (ZrCxN1-x) coating embedded with silver nanoparticles or hydrogenated diamond-like carbon (DLCH) coating. They have shown good performance on the wear resistance, hardness, and biocompatibility. The other techniques, like ion beam surface modification, photolithography, nanoimprint lithography, and laser surface texturing, are also being explored in the industry [44].

While we look optimistically at the future, we should never forget our past failures where several attempts have been made to reinforce PE's mechanical properties. A few unsuccessful attempts are carbon-fiber-reinforced poly (CFR-UHMWPE) marketed as Poly II (Zimmer) and Hylamer (Depuy). The Poly II was introduced in 1970 but was discontinued due to the rupture of the surface fibers and reduced crack resistance than the virgin UHMWPE [35]. In the late 1980s, high-pressure recrystallized poly was released as Hylamer, which demonstrated higher susceptibility to oxidation than the virgin UHMWPE. Its poor performance was attributed to gamma sterilization in the air and fell out of favor in the late 1990s with the development of XLPE [49].

## Conclusions

There are several ways to process PE and manipulate their mechanical properties, wear characteristics, and oxidative resistance. An ideal PE’s properties include strong resistance to wear, oxidation, and FCP and should retain these properties in-vivo for the long-term. Wear resistance is improved by crosslinking the PE, which is accomplished by higher doses of radiation (Gamma or E-beam). However, such radiation doses generate more free radicals that promote long-term oxidative degradation of PE. Post-irradiation thermal treatment (first-generation XLPE) can quench these free radicals improving oxidative stability but at the expense of compromising the mechanical properties, especially fatigue resistance. Sequential irradiation-annealing (X3) improves mechanical properties while preserving the wear properties, although there is a theoretical concern about long-term oxidation in-vivo. Instead of thermal treatment, incorporating antioxidants or sequential irradiation constitutes a balanced environment to create oxidation- and fatigue-resistant PE while maintaining its wear characteristics. Despite all newer developments and laboratory studies to date, there is no conclusive evidence that HXLPE would improve clinical outcomes when compared to CPE in TKR. The manufacturers have utilized different PE processing methods, which can influence PE’s long-term performance. Surgeons are encouraged to critically look for specific failure mechanisms based on these processing techniques. 
